# Potential value of gastrointestinal myoelectrical activity in the diagnosis of anxiety-depression disorder: a population-based study

**DOI:** 10.1186/s12888-023-05319-1

**Published:** 2023-11-29

**Authors:** Baichuan Li, Anjiao Peng, Danxuan Yang, Na Yang, Xia Zhao, Peimin Feng, Zhenlei Wang, Lei Chen

**Affiliations:** 1 Department of Neurology, West China Hospital, Joint Research Institution of Altitude Health, Sichuan University, Chengdu, 610041 China; 2 Department of Clinical Research Management, West China Hospital, Sichuan University, Chengdu, 610041 China; 3 Department of Gastroenterology, Hospital of Chengdu University of Traditional Chinese Medicine, Chengdu, 610032 China; 4 Clinical Trial Center, NMPA Key Laboratory for Clinical Research and Evaluation of Innovative Drug, West China Hospital, Sichuan University, Chengdu, 610041 China; 5 Department of Frontiers Science Center for Disease-related Molecular Network, West China Hospital, Sichuan University, Chengdu, 610041 China

**Keywords:** Depression, Anxiety, Electrogastroenterogram

## Abstract

**Background:**

Depression and anxiety are frequently coexisted mental illness. The lack of solid objective diagnostic criteria has led to a high rate of suicide. The brain-gut axis bridges the gastrointestinal system with neuropsychiatric disorders. However, it is still not possible to reflect mental disease with gastrointestinal information. The study aimed to explore the auxiliary diagnostic value of gastrointestinal myoelectrical activity in anxiety-depression disorders (ADD) without gastrointestinal disturbance.

**Methods:**

A natural population cohort from 3 districts in Western China were established. The Patient Health Questionnaire-9 and the Generalized Anxiety Disorder-7 were used to assess ADD. Gastrointestinal myoelectrical activity of ADD were measured by multi-channel cutaneous electrogastroenterogram (EGEG). Then the parameters of EGEG between ADD and healthy controls were analyzed.

**Results:**

The average amplitude and response area of intestinal channel in ADD were significantly lower than those of controls (153.49 ± 78.69 vs. 179.83 ± 103.90, 57.27 ± 29.05 vs. 67.70 ± 38.32), which were shown to be protective factors for ADD (OR = 0.944 and 0.844, respectively). Further, the scale item scores related to the core symptoms of anxiety and depression were also associated with these two channels (*p* < 0.05), and the gastrointestinal electrical signals of ADD are significantly changed in the elderly compared to the young adults.

**Conclusions:**

The intestinal myoelectrical activity has a certain auxiliary diagnostic value in psychiatric disorders and is expected to provide objective reference for the diagnosis of anxiety and depression.

**Supplementary Information:**

The online version contains supplementary material available at 10.1186/s12888-023-05319-1.

## Background

Anxiety and depression disorders are major global mental illness, ranking as the third cause of disease burden worldwide [1]. Comorbidity anxiety and depression is very common [2, 3], which increased the suicide risk 20 times than that of the normal population [4]. The gold standard for mental illness diagnosis is mainly based on the scale scores, but scale assessment is time consuming and has a great degree of subjectivity, and many participants are unable to complete the questionnaire effectively due to cultural differences and literacy level, which has become a bottleneck limiting the precise treatment of mental diseases. Although there are studies that have used machine learning methods for the adjunctive diagnosis of anxiety and depression, and obtained good accuracy [5]; however, most of the current prediction use supervised model, which require inputting the expert judgment or patient self-reported data [6]. As a result, the prediction results of the model are still affected by subjective judgment, which limits the clinical application of the method. Therefore, it is of great significance to explore objective auxiliary diagnostic methods and improve the diagnosis rate of anxiety and depression in order to reduce the suicide rate and improve the prognosis of patients.

In recent years, both clinical and basic research have found that mental diseases are associated with disturbance of gastrointestinal function. Most patients with functional dyspepsia are accompanied by major depressive disorder; and in patients with generalized anxiety disorder, symptoms of impaired gastrointestinal function such as loss of appetite and indigestion, are often present [7, 8]. The underlying mechanism may be a dysbiosis of the intestinal flora or a disruption of the enteric nervous system, etc. [9, 10].

Cutaneous electrogastroenterogram (EGEG) testing has become a routine tool for clinical screening of gastrointestinal disorders, which is of convenient, high accuracy, low cost, time saving, non-invasive, and provides a more objective reflection of gastrointestinal myoelectrical activity through the detection of multipoint electrical signals [11]. Previous studies have used EGEG as an aid to the diagnosis of functional dyspepsia combined with depression and anxiety [12], but it has not been used to assess single depressive and anxiety disorders without gastrointestinal discomfort.

Therefore, we used a novel eight-channel EGEG device to record gastrointestinal myoelectrical activity among a natural population cohort from three districts about 60 community in Western China, and found that EGEG is diagnostic for patients with single anxiety and depression but no gastrointestinal disorders. This study provides an objective basis for the diagnosis of anxiety and depression, and is expected to make the EGEG examination a supplement and alternative for the scale assessment.

## Methods

### Participants and study design

A natural population cohort was established from three administrative regions (Longquan, Mianzhu, Pidu) in Western China, aged 18 years or older, was recruited to collect general information on participants’ age, gender, marital status, education level, lifestyle, and dietary habits, to carry out the assessment of psychological scale, and to perform EGEG test. All participants voluntarily participated in the study and signed an informed consent form.

Participants with a recent diagnosis (within the preceding six months) of gastrointestinal disorders such as gastritis or gastric ulcers, or those experiencing gastrointestinal discomfort (e.g., diarrhea, constipation), as well as individuals with severe organ dysfunction or metabolic conditions like diabetes mellitus, were excluded from the study. To mitigate potential confounding effects of medication on the EGEG test results, individuals who had taken any medications within one month prior to the examination were also excluded. This study was approved by the Ethics Committee of West China Hospital of Sichuan University (No.2018 − 491 and 2022 − 697).

The research was a cross-sectional observational study. The primary endpoint was the occurrence of depression and anxiety, while the secondary endpoint encompassed the variation in gastrointestinal electrical signals among individuals experiencing depression and anxiety, stratified by both age and gender.

### Mental state assessment

The Patient Health Questionnaire-9 (PHQ-9) was used to assess the depressive state and the Generalized Anxiety Disorder-7 (GAD-7) was used to assess the anxiety state. Both scales have good reliability and validity and are completed under the guiding of two professionals in a quiet environment, using unified terminology. Given the comorbidity of anxiety and depression is very common, participants diagnosed with either anxiety or depression were included as anxiety-depression disorder (ADD) in this study.

### EGEG records

**Fig. 1:**
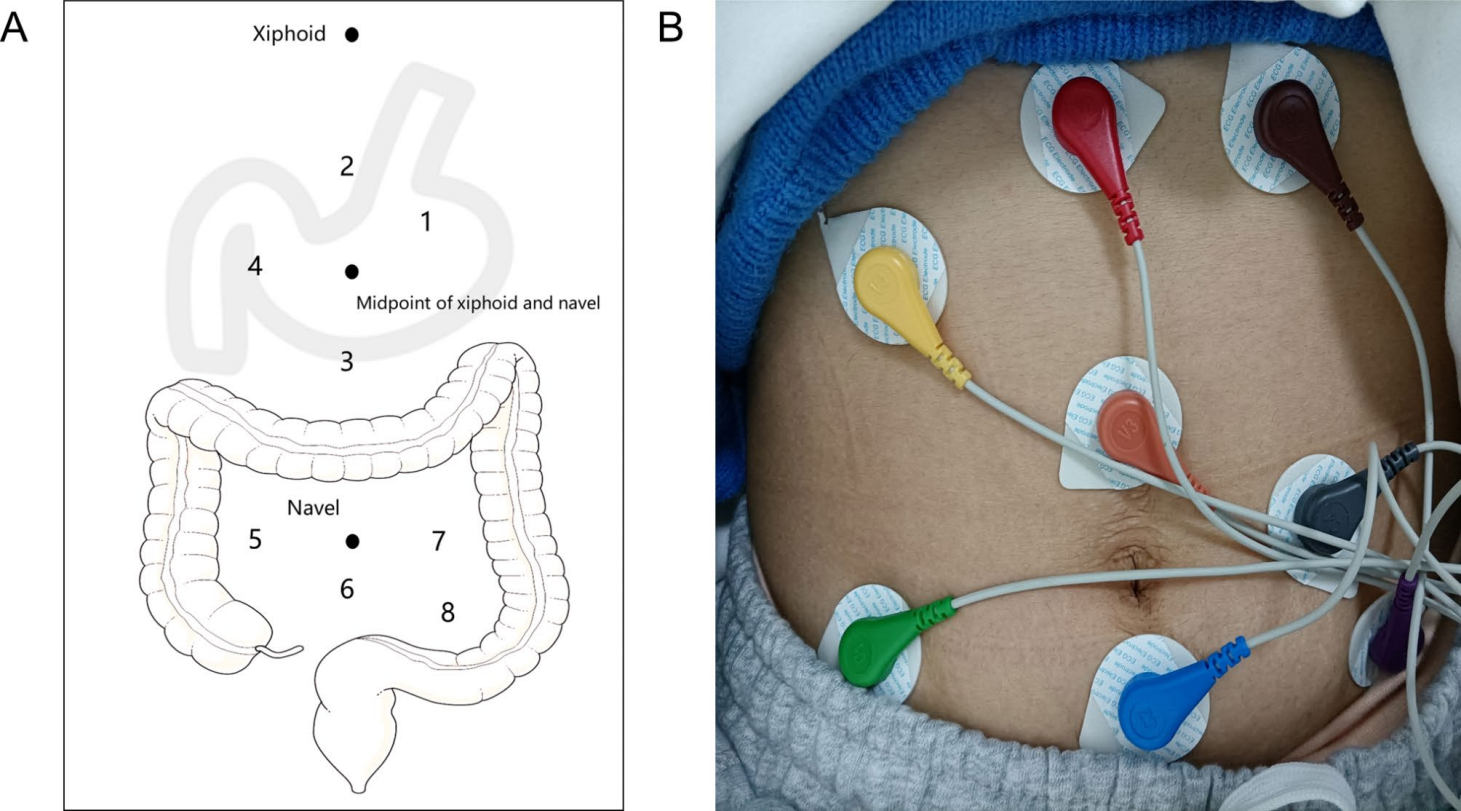
(**A**) The pattern diagram of electrodes position of EGEG recording. And eight electrodes represent eight channels to reflect the myoelectrical activity of corpus gastricum, lesser curvature, greater curvature, antrum, ascending colon, transverse colon, descending colon and rectum. (**B**) The diagram of process in EGEG examination. EGEG, Electrogastroenterogram

Gastrointestinal myoelectrical activity was measured and collected with an eight-channel gastrointestinal electromyograph (XDJ-S8, Hefei Kaili Co., Hefei, China). All subjects were instructed to avoid alcohol and spicy foods (e.g., chili, pepper, ginger, mustard) for three days and to fast for at least 6 h before the examination. Measurements were performed in the supine position (Fig. 1). Four gastric electrodes and four intestinal electrodes were placed on the abdominal skin (Hanjie Co. Ltd., Shanghai, China). During the examination, the subject was instructed to avoid any movement or talk. Considering that the position of the stomach and intestine will have a large movement after the meal, only the pre-meal data of each electrode were recorded in the analysis.

### Gastrointestinal electrical index

The EGEG sampling rate was 1 Hz and the filtering frequency was 0.008-0.1 Hz to filter out the background noise including the heartbeat. After detecting the artifacts, the raw EGEG potential data were calculated by the software accompanying the checker, and the following parameters were derived through the software for spectral analysis. (1) mean amplitude (MA); (2) mean frequency (MF); (3) percentage of disturbed electrical rhythm (PDER); (4) response area (RA); (5) dominant frequency (DF); (6) dominant power (DP); (7) percentage of normal slow wave (PNSW); and (8) slow wave frequency instability coefficient (SWFIC). Observations with values of gastric electrical parameters greater than outside five times the standard deviation of their mean were considered as data abnormalities, and the subject was considered for exclusion during the analysis.

### Statistical analysis

Data analysis was performed using SPSS 26.0, and the measurement data were expressed as mean ± standard deviation (Mean ± SD) and the count data were expressed as [number of cases, %]. The Kolmogorov-Smirnov test was used to assess the normality of the samples. Student’s t-tests were used when normally distributed measures were satisfied; Mann-Whitney U-tests were used when normally distributed measures were not satisfied. Chi-square analysis was used for the comparison of count data, and Fisher’s exact test was used for theoretical frequencies less than five. The indicators with differences between the two samples were selected and the correlation analysis was carried out with mental-psychological scores. The Pearson correlation analysis was used if they met normal distribution and chi-square, otherwise, the Spearman analysis was used. The multivariate logistic regression analysis was conducted. *p* < 0.05 was considered a statistically significant difference.

## Results

### Clinical baseline information of participants

**Fig. 2:**
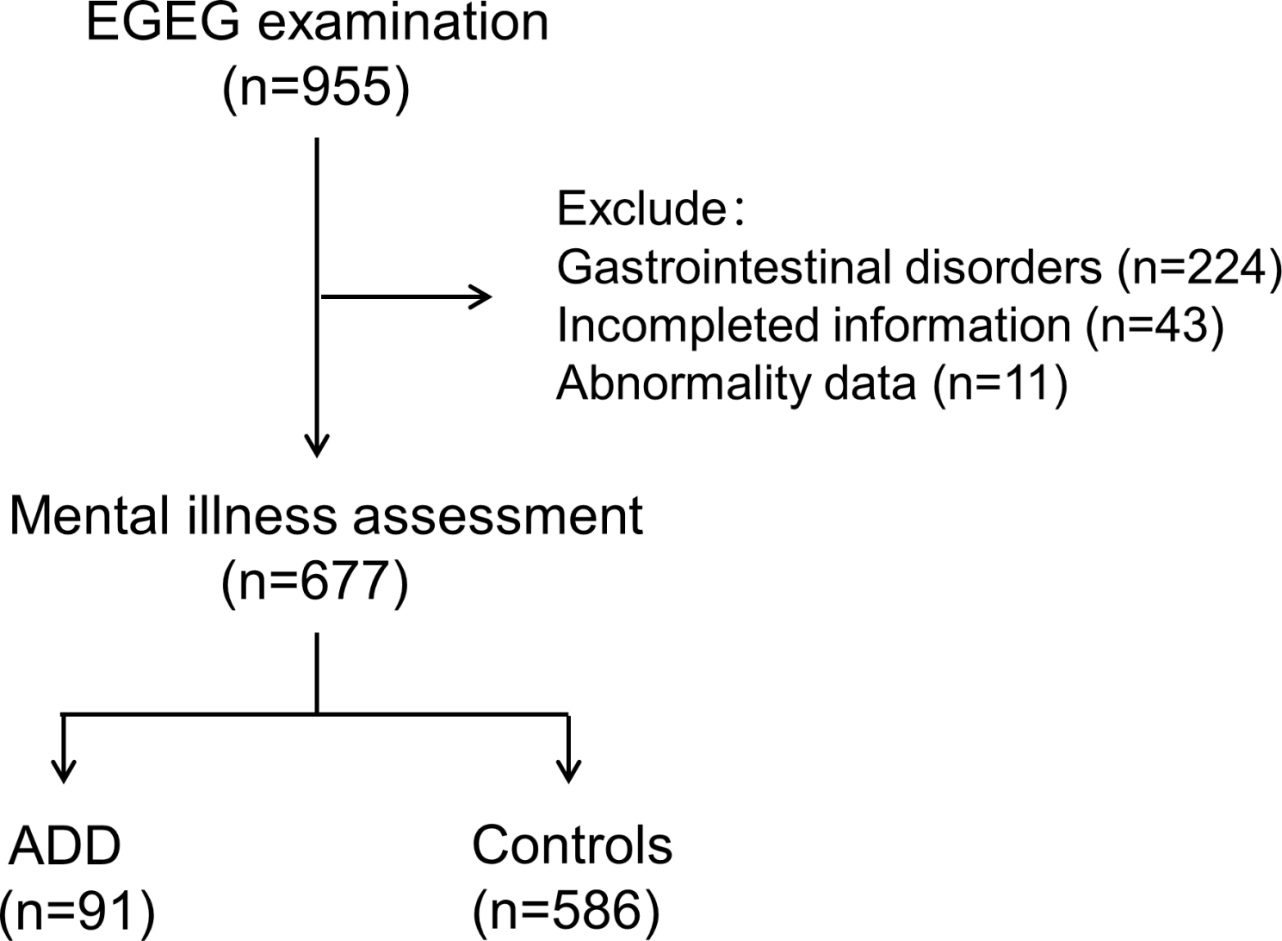
The flowchart of participant enrollment

A total of 955 cases in the natural population cohort completed gastrointestinal electrogram testing, excluding 224 cases with gastrointestinal disorders or discomfort such as diarrhea or constipation within six months, 43 cases with incomplete basic information, and 11 cases with abnormal values. The remaining 677 cases completed the depression and anxiety scale assessment, of which 91 cases (13.4%) had anxiety and depression disorders and 586 cases (86.6%) were healthy people. General information was compared in Table 1, and the recruitment process was shown in Fig. 2.

**Table 1:** The demographic and clinical baseline of ADD and Controls

	ADD (n = 91)	Controls (n = 586)	Test value	P
Age	56.48 ± 6.70	55.87 ± 6.66	0.819 ^a^	0.309
Gender (male, %)	21(23.08%)	185(31.57%)	2.684^c^	0.101
Body mass index	23.71 ± 2.92	24.35 ± 3.11	1.817 ^a^	0.070
Smoke (yes, %)	11(12.09%)	77(13.14%)	0.077 ^c^	0.781
Alcohol (yes, %)	14(15.38%)	148(25.26%)	4.217 ^c^	0.040
PHQ-9 score	5.66 ± 4.49	0.6 ± 1.15	-14.354 ^b^	**< 0.001**
GAD-7 score	5.87 ± 3.83	0.37 ± 0.88	-16.340 ^b^	**< 0.001**
Glucose	5.28 ± 1.11	5.52 ± 1.39	1.577 ^a^	0.115
Triglyceride	1.52 ± 0.80	1.65 ± 1.38	0.865 ^a^	0.387
Total cholesterol	5.38 ± 1.02	5.38 ± 0.94	0.018 ^a^	0.985
High-density lipoprotein	1.78 ± 0.51	1.75 ± 0.49	0.544 ^a^	0.587
Low-density lipoprotein	3.00 ± 0.74	3.01 ± 0.69	0.060 ^a^	0.952

Note: ^a^ refers to ‘t’ value from Student’s t-tests; ^b^ refers to ‘Z’ value from Mann-Whitney U-tests; ^c^ refers to ‘X^2^’ value from Chi-square analysis. ADD, anxiety-depression disorders

### Abnormal intestinal myoelectrical activity in the ADD

**Fig. 3:**
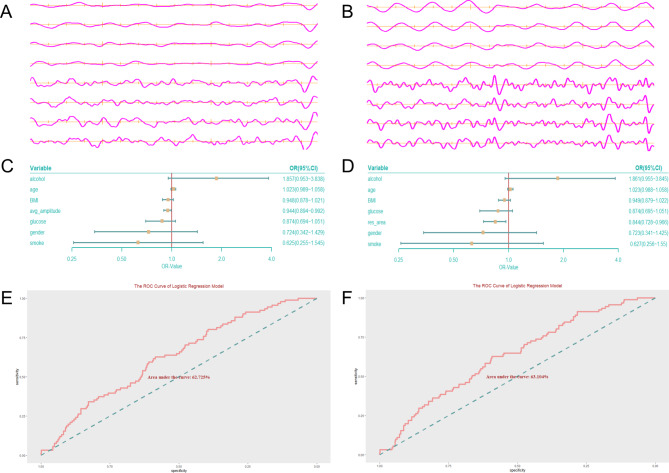
The EGEG oscillogram of ADD (**A**) and healthy participants (**B**). The regression forest model of mean amplitude (**C**) and response area (**D**) of intestinal channel. (**E-F**) Receiver operating curve analysis of two regression model. EGEG, Electrogastroenterogram; ADD, anxiety-depression disorder

As seen in the EGEG of both groups, the amplitude of each channel was significantly lower in the ADD than in the healthy participants (Fig. 3A, B). In the most previous studies, merged channels were used to reflect each position of gastro/intestinal myoelectrical activity. We performed the correlation analysis of each parameter between channels one to four and channels five to eight at first (Supplementary Table 1), and the results showed that there was a high correlation between four channels in the gastric region and four channel in the intestinal region, so we considered merging channel one to four as gastro channel and channel five to eight as intestinal channel.

Then, the signal differences between the gastric and intestinal channels were compared in the two groups. The results showed that there was no significant difference in the parameters of the gastric channel compared with those of the two groups of participants. However, in the intestinal channel, the MA and RA were lower in ADD compared to the healthy participants (*p* < 0.05) (Table 2).

**Table 2:** Comparison of EGEG parameters in gastric and intestinal channel between ADD and controls

	Parameters	ADD (n = 91)	Controls (n = 586)	Test value	*P*
Gastro channel	Mean amplitude	158.68 ± 73.43	172.64 ± 83.15	-1.389 ^b^	0.165
	Mean frequency	3.43 ± 0.28	3.46 ± 0.31	-0.789 ^b^	0.430
	Percentage of disturbed gastric electrical rhythm	20.86 ± 3.7	20.94 ± 3.7	1.553 ^a^	0.121
	Response area	56.8 ± 25.77	61.93 ± 29.83	-1.319 ^b^	0.187
	Dominant frequency	2.98 ± 0.20	3 ± 0.25	-0.720 ^b^	0.471
	Dominant power	62.52 ± 5.43	61.42 ± 6.25	1.597 ^a^	0.111
	Percentage of normal gastric slow wave	61.02 ± 7.31	59.32 ± 8.33	1.840 ^a^	0.066
	Slow wave frequency instability coefficient	1.94 ± 0.79	1.98 ± 0.8	-0.499 ^b^	0.618
Intestinal channel	Mean amplitude	153.49 ± 78.69	179.83 ± 103.90	**-1.941** ^b^	**0.050**
	Mean frequency	12.46 ± 2.03	12.80 ± 2.34	-1.133 ^b^	0.257
	Percentage of disturbed intestinal electrical rhythm	23.94 ± 5.10	23.98 ± 5.53	-0.134 ^b^	0.893
	Response area	57.27 ± 29.05	67.70 ± 38.32	**-2.119** ^b^	**0.034**
	Dominant frequency	11.24 ± 2.37	11.60 ± 2.76	-0.831 ^b^	0.406
	Dominant power	30.26 ± 5.79	30.47 ± 5.85	-0.548 ^b^	0.584
	Percentage of normal intestinal slow wave	53.93 ± 10.26	52.57 ± 12.39	-0.900 ^b^	0.368
	Slow wave frequency instability coefficient	0.16 ± 0.09	0.16 ± 0.10	-0.274 ^b^	0.784

Note: ^a^ refers to ‘t’ value from Student’s t-tests; ^b^ refers to ‘Z’ value from Mann-Whitney U-tests. ADD, anxiety-depression disorders

### MA and RA of intestinal channel as protective factors for the ADD

Correlation analysis of the MA and RA found above revealed a strong correlation between the two parameters (r = 0.995, *p* < 0.001), so in logistic regression analysis, the two parameters were taken to the model with the covariates separately (Table 3). Alcohol consumption, a factor that was found to be different between two groups at the univariate analysis, was not significantly difference in the regression models. After the covariates were adjusted, the results showed that both MA and RA of the intestinal channel were protective factors for developing ADD (OR = 0.944 and 0.844, respectively; Fig. 3C-D). Receiver operating curve of logistic regression exhibited that the area under curve was 62.725% and 63.104% respectively, which reflected the performance of the models were great (Fig. 3E-F).

**Table 3:** Regression analysis of gastrointestinal electrical signaling and ADD.

	Factors	*B*	*SE*	*Wald*	*P*	*OR*
Model 1	Gender	-0.322	0.363	0.790	0.374	0.724
	Age	0.023	0.017	1.689	0.194	1.023
	Smoke	-0.470	0.455	1.069	0.301	0.625
	Alcohol	0.619	0.354	3.061	0.080	1.857
	Body mass index	-0.053	0.038	1.919	0.166	0.948
	Glucose	-0.135	0.105	1.651	0.199	0.874
	Mean amplitude of intestinal channel	0.058	0.026	4.822	**0.028**	**0.944**
Model 2	Gender	-0.325	0.363	0.802	0.370	0.723
	Age	0.022	0.017	1.655	0.198	1.023
	Smoke	-0.467	0.455	1.055	0.304	0.627
	Alcohol	0.621	0.353	3.087	0.079	1.861
	Body mass index	-0.052	0.038	1.834	0.176	0.949
	Glucose	-0.134	0.105	1.645	0.200	0.874
	Response area of intestinal channel	-0.170	0.072	5.553	**0.018**	**0.844**

Note: ADD, anxiety-depression disorders

### Core symptoms of the ADD are associated with abnormal intestinal channel signaling

The PHQ-9 and GAD-7 have high reliability and validity and are of good diagnostic value for depression and anxiety. The core item of PHQ-9 is related to depression, i.e., item 9 (thoughts of being better off dead or hurting oneself in some way); the core item of GAD-7 is related to worry, i.e., item 5 (inability to sit still due to restlessness). Then, we selected the core items of the two scales for correlation analysis with the differential intestinal channel parameters. The results showed that item 9 of PHQ-9 was correlated with MA and RA of the intestinal channels (r=-0.091, *p* = 0.018; r=-0.096, *p* = 0.013), and item 5 of GAD-7 was also correlated with two parameters (r=-0.076, *p* = 0.049; r=-0.078, *p* = 0.042).

### Intestinal channel abnormalities have greater diagnostic significance for ADD in the elderly

Since Gastrointestinal myoelectrical activity have the role of auxiliary judgment in mental illness, we then explored its diagnostic significance for different gender and age groups. As for the study of different genders, the results demonstrated that women were similar to men, and there were no significant differences in all channel parameters between those with ADD and healthy participants. About ages, we divided the subjects into elderly (> 60 years old) and young adults (18–60 years old) according to the WHO criteria. We found that the MA and RA of those suffering from ADD were significantly lower in the elderly than in the healthy participants (*p* < 0.05), while in the young adult, there was no significant difference between the two groups compared to each parameter (Table 4).

**Table 4:** Comparison of gastrointestinal electrical signaling of ADD in different genders and ages

		MA of intestinal channel	Z	P	RA of intestinal channel	Z	P
Gender							
Male	ADD	148.39 ± 74.75	-1.383	0.167	55.88 ± 28.65	-1.433	0.152
	Controls	186.02 ± 107.14			69.30 ± 39.02		
Female	ADD	155.01 ± 80.30	-1.376	0.169	57.69 ± 29.37	-1.587	0.113
	Controls	176.98 ± 102.38			66.97 ± 38.02		
Age							
Elderly	ADD	120.58 ± 52.34	**-3.515**	**< 0.001**	45.98 ± 19.72	**-3.260**	**0.001**
	Controls	185.49 ± 99.97			69.14 ± 36.63		
Young adults	ADD	165.95 ± 83.60	-0.319	0.750	61.55 ± 30.95	-0.636	0.525
	Controls	178.3 ± 105.00			67.31 ± 38.79		

Note: ADD, anxiety-depression disorders; MA, mean amplitude; RA, response area

## Discussion

The current diagnosis of anxiety, depression and other psychiatric disorders is mainly based on scale scores. However, scale assessment is highly subjective, and some subjects have limited literacy and are unable to accurately understand the content of the scales. In addition, most of the scales applied originate from the West, and when translated, they may not be able to effectively convey the meaning of the original scales due to textual differences, resulting in lower scale reliability and validity [13]. These factors will greatly affect the effectiveness of scale measurement and disease diagnosis and treatment, thus increasing patient mortality and socio-economic burden. In this study, we selected a multichannel EGEG to objectively record the gastrointestinal myoelectrical activity of a natural population with no gastrointestinal discomfort within 6 months and no history of drug use within 1 week, and found that the mean amplitude and response area of the gastrointestinal electrical signals, especially in the intestinal channel, could effectively reflect anxiety and depression disorders.

EGEG, similar to electrocardiograph (ECG) and electroencephalogram (EGG), is a method of recording gastrointestinal myoelectrical activity on the surface of abdominal surface using skin electrodes, which is convenient, non-invasive and objective [14]. Because the gastrointestinal electrical signal is considerately weak and slow, the amplitude of which is only 1/1000 of that of ECG, the research on EGEG has started rather late and developed slowly [15]. EGEG have now been used as a routine clinical aid in diagnosis. A large number of studies have shown that EGEG has good diagnostic role in gastrointestinal diseases such as gastritis, gastric ulcer, tachycardia/bradycardia, etc. It also has some diagnostic significance for functional dyspepsia with obesity, motion sickness, post-surgical gastrointestinal dysfunction [16–18]. Although worldwide researchers have figured out certain rules for the clinical research of electrogastrogram, some criteria have been developed for the auxiliary diagnosis of diseases, such as low amplitude (< 150µV) for gastritis, high amplitude (> 250µV) and high frequency (> 3.5CPM) for gastric ulcer. However, there is still a lack of unified expert consensus on the study of intestinal electrograms, and researchers are still trying to explore and summarize them in depth [19, 20]. This study for the first time found that the diagnostic significance of intestinal electrograms for mental illness is higher than that of gastric electrograms, which will also further contribute to the development of intestinal electrograms and promote the formation of a standardized interpretation scheme and a more scientific and effective delineation of critical values.

Moreover, considering the potential variation in the anatomical positioning of the stomach and intestines among subjects, we addressed this concern by employing a multichannel gastrointestinal electrography instrument. This instrument enabled the collection of gastrointestinal myoelectrical activity from four distinct sites in both the stomach and intestines. Subsequently, through correlation analysis, we observed a substantial correlation between channels one to four in the stomach and channels five to eight in the intestines. Consequently, we opted for channel integration as a method to substitute the gastrointestinal myoelectrical activity, utilizing the combined parameters from both gastric and intestinal channels. This approach served to mitigate potential result biases stemming from inter-individual organ positioning discrepancies.

Although EGEG have been reported to be associated with depression and anxiety in previous studies, most of them have been applied to patients with gastrointestinal disorders, such as observing the improvement effect on depression and anxiety by relieving gastrointestinal discomfort. It has not been directly investigated whether EGEG can be used as an objective diagnostic tool for mental disease. Therefore, the present study provides strong evidence that abnormality gastrointestinal myoelectrical activity is observed among participants of anxiety and depression without gastrointestinal dysfunction, as evidenced by a decrease in the mean amplitude and response area.

According to epidemiological data, the prevalence of depression is about 1.5-3 times higher in women than in men [21, 22], and the gender distribution of the subjects in this study showed that women are about 3 times more likely to be depressed than men, which is consistent with the disease characteristics. In addition, in the traditional diagnosis of depression and anxiety, the depression self-rating scale, anxiety self-rating scale, and Hamilton anxiety scale are often selected, but these scales have more questions and complicated items, which are not conducive to use in natural population cohorts [23]; in contrast, the PHQ-9 and GAD-7 selected in this study have fewer questions, condensed items, and high reliability, which are more effective in cohort screening. After we found depression and anxiety judged on the basis of total scale scores were correlated with EGEG, the core items of the scale that reflect the core symptoms of depression and anxiety were also correlated with gastrointestinal electrical parameters (r > 0, *p* < 0.05), further suggesting that EGEG can objectively reflect anxiety-depression disorder.

On this basis, we further classified the population according to different genders (male/female) and different ages (over 60 years old for elderly and 18–60 years old for young adults). We found that females were similar to males, and there were no significant differences in all channel parameters between those with ADD and healthy participants. The MA and RA of those with ADD were significantly lower in the elderly than in the healthy control population (*p* < 0.05), while in young adult subjects, there was no significant difference between the two groups compared to each parameter. This shows that EGEG can not only reflect anxiety-depression disorders, but also have a significantly higher diagnostic value in the elderly than in young adults.

Nevertheless, the study included several limitations. Although the results came from multi-centers of Western China, the sample size was still relatively small. Expanded size and incorporated participants in other areas are essential in the future. Furthermore, this was a cross-sectional study and only measured gastrointestinal myoelectrical activity once, the causality could not be drawn. In future studies, the gastrointestinal electrical activity and mental state of the participants at multiple time points can be measured through follow-up, which is helpful to establish the dynamic relationship between gastrointestinal electrical activity and anxiety-depression disorder.

## Conclusion

In conclusion, in this study, by comparing and analyzing the gastrointestinal myoelectrical activity between ADD and healthy participants, it was found that the mean amplitude and response area of intestinal channel in ADD were significantly lower than those of healthy subjects, suggesting that intestinal myoelectrical activity have some auxiliary diagnostic value in psychiatric disorders and are more significant in the elderly. This study is expected to provide objective reference for the diagnosis of depression and anxiety, thus improving the diagnosis, reducing the suicide rate, as well as reducing the impact and burden on patients and society.

### Electronic supplementary material

Below is the link to the electronic supplementary material.

Supplementary Material 1 Table 1

## Data Availability

The dataset supporting this article’s conclusions is available with a request to the corresponding author (Lei Chen).

## Abbreviations

EGEG: Electrogastroenterogram
PHQ-9: Patient Health Questionnaire-9
GAD-7: Generalized Anxiety Disorder-7
ADD: Anxiety-depression disorder
MA: Mean amplitude
MF: Mean frequency
PDER: Percentage of disturbed electrical rhythm
RA: Response area
DF: Dominant frequency
DP: Dominant power
PNSW: Percentage of normal slow wave
SWFIC: Slow wave frequency instability coefficient
ECG: Electrocardiograph
EGG: Electroencephalogram
