# Supplementary material for: Potential value of gastrointestinal myoelectrical activity in the diagnosis of anxiety-depression disorder: a population-based study
Source: BMC Psychiatry. 2023 Nov 29;23:891. doi: 10.1186/s12888-023-05319-1 (PMC10685574; doi:10.1186/s12888-023-05319-1)
Supplement: Supplementary file 1 — Supplementary Material 1 Table 1 [file 12888_2023_5319_MOESM1_ESM.docx]

**Supplementary Table1.** Correlation analysis of gastrointestinal electrical signaling of each EGEG channel.

|  | MA | MF | PDER | RA | DF | DP | PNSW | SWFIC |
| --- | --- | --- | --- | --- | --- | --- | --- | --- |
| Channel 1 vs 2 | 0.814 | 0.622 | 0.437 | 0.832 | 0.361 | 0.670 | 0.765 | 0.479 |
| Channel 1 vs 3 | 0.816 | 0.548 | 0.349 | 0.836 | 0.328 | 0.634 | 0.751 | 0.409 |
| Channel 1 vs 4 | 0.753 | 0.547 | 0.352 | 0.787 | 0.275 | 0.531 | 0.646 | 0.358 |
| Channel 2 vs 3 | 0.827 | 0.595 | 0.366 | 0.847 | 0.307 | 0.638 | 0.748 | 0.483 |
| Channel 2 vs 4 | 0.825 | 0.562 | 0.375 | 0.849 | 0.360 | 0.620 | 0.714 | 0.439 |
| Channel 3 vs 4 | 0.820 | 0.612 | 0.375 | 0.844 | 0.350 | 0.676 | 0.743 | 0.500 |
| Channel 5 vs 6 | 0.893 | 0.861 | 0.746 | 0.905 | 0.697 | 0.807 | 0.915 | 0.748 |
| Channel 5 vs 7 | 0.902 | 0.826 | 0.716 | 0.912 | 0.673 | 0.764 | 0.877 | 0.675 |
| Channel 5 vs 8 | 0.904 | 0.805 | 0.701 | 0.912 | 0.637 | 0.764 | 0.877 | 0.672 |
| Channel 6 vs 7 | 0.897 | 0.856 | 0.736 | 0.906 | 0.665 | 0.794 | 0.895 | 0.712 |
| Channel 6 vs 8 | 0.884 | 0.834 | 0.674 | 0.893 | 0.655 | 0.790 | 0.884 | 0.667 |
| Channel 7 vs 8 | 0.928 | 0.865 | 0.737 | 0.934 | 0.669 | 0.849 | 0.934 | 0.747 |

Note: The results presented in the table were correlation coefficients and each comparison was significantly difference (*p*<.001). MA, mean amplitude; MF, mean frequency; PDER, percentage of disturbed electrical rhythm; RA, response area; DF, dominant frequency; DP, dominant power; PNSW, percentage of normal slow wave; SWFIC, slow wave frequency instability coefficient.
